# End-tidal carbon dioxide monitoring may be associated with a higher possibility of return of spontaneous circulation during out-of-hospital cardiac arrest: a population-based study

**DOI:** 10.1186/s13049-015-0187-y

**Published:** 2015-11-24

**Authors:** Jiun-Jia Chen, Yi-Kung Lee, Sheng-Wen Hou, Ming-Yuan Huang, Chen-Yang Hsu, Yung-Cheng Su

**Affiliations:** School of Medicine, Tzu Chi University, Hualien, Taiwan; Emergency Department, Dalin Tzu Chi Hospital, Buddhist Tzu Chi Medical Foundation, No. 2, Minsheng Rd., Dalin Township, Chiayi County 622, Taiwan, R.O.C.; Emergency Department, Shin-Kong Wu Ho-Su Memorial Hospital, Taipei, Taiwan; Department of Emergency Medicine, Mackay Memorial Hospital, Taipei, Taiwan; Department of Public Heath, National Taiwan University, Taipei, Taiwan

**Keywords:** End-tidal carbon dioxide, Cardiac arrest, Out-of-hospital cardiac events, Capnography

## Abstract

**Background:**

During cardiac arrest, end-tidal carbon dioxide (ETCO_2_) monitoring is recommended as a chest compression performance indicator. However, its frequency of use during out-of-hospital cardiac arrest (OHCA) and its benefits have never been evaluated in real clinical situations.

**Objective:**

We investigated OHCA patients in Taiwan to evaluate the frequency of ETCO_2_ monitoring and its effects on sustained return of spontaneous circulation (ROSC).

**Methods:**

We sampled the Taiwan National Health Insurance claims database, which contains 1 million beneficiaries. All adult beneficiaries older than 18 years who presented with OHCA and received chest compression between 1 January 2005 and 31 December 2012 were enrolled. We further identified patients with ETCO_2_ monitoring and matched each 1 with 20 patients who did not receive ETCO_2_ monitoring based on their propensity scores. A simple conditional logistic regression model was applied to compare the odds ratio (OR) for sustained ROSC in the matched cohorts.

**Results:**

A total of 5041 OHCA patients were enrolled. The frequency of ETCO_2_ monitoring has increased since 2010 but still is low. After matching, 53 patients with ETCO_2_ monitoring and 1060 without ETCO_2_ monitoring were selected. The OR of sustained ROSC in the ETCO_2_ group was significantly increased (2.38, 95 % CI 1.28–4.42).

**Conclusion:**

Patients who received ETCO_2_ monitoring during OHCA had a higher possibility of sustained ROSC, but the overall use of ETCO_2_ monitoring is still low despite strong recommendations for its use.

**Electronic supplementary material:**

The online version of this article (doi:10.1186/s13049-015-0187-y) contains supplementary material, which is available to authorized users.

## Introduction

Out-of-hospital cardiac arrest (OHCA) is a major cause of morbidity and mortality around the world. In the United States, the estimated annual incidence of OHCA ranges from 300,000 to 350,000 each year [1, 2]. Wider application of the improved algorithms in the Advanced Cardiac Life Support (ACLS) recommendations, such as early defibrillation and high-quality cardiopulmonary resuscitation (CPR), have led to increased survival rates among OHCA patients [3–5]. Because CPR consistency is an important factor, end-tidal carbon dioxide (ETCO_2_) monitoring has recently been suggested as an adjunctive tool for monitoring the effectiveness of chest compressions during resuscitations [3, 6].

The height of the ETCO_2_ level during CPR is well correlated with cardiac output during chest compression [7–9], and feedback about an inadequate level can help the team to evaluate possible causes such as misplaced or displaced tracheal tube, fatigue of the team member, suboptimal chest compressions, cardiac tamponade, or pneumothorax. In this way the resuscitation can be individualized, and better CPR delivery may be achieved. However, to our knowledge, despite the strong recommendations from ACLS, the frequency of ETCO_2_ use during OHCA and its possible influence on CPR quality and survival, have not been evaluated in population-based studies.

In this study, a large administrative database was used to assess sustained ROSC in OHCA patients who were treated with ETCO_2_ monitoring in Taiwan. We hypothesized that use of ETCO_2_ monitoring may be associated with a higher possibility of sustained ROSC because of real-time feedback about the quality of chest compression. The results of this study may help clinicians to place more emphasis on the use of ETCO_2_ monitoring in frequently encountered situations.

## Methods

### Ethics statement

This study was initiated after its protocol was approved by the Institutional Review Board of Dalin Tzu Chi Hospital, Buddhist Tzu Chi Medical Foundation, Taiwan, and was conducted in conformity with the Declaration of Helsinki.

### Database

The National Health Insurance (NHI) program was implemented in Taiwan in 1995 and provides compulsory universal health insurance. It enrolls about 99 % of the Taiwanese population and contracts with 97 % of all the country’s medical providers [10, 11]. The database contains comprehensive information about all insured subjects, including sex, date of birth, residential or work area, dates of clinical visits, diagnoses identified by International Classification of Diseases (Ninth Revision) Clinical Modification (ICD-9-CM) diagnostic codes, details of prescribed medications and procedures administered, expenditure amounts, and outcome at hospital discharge (i.e., recovered, died, or transferred out) [12]. A random sample of 1,000,000 people who received health benefits from the NHI program was selected based on calendar-year 2005 reimbursement data and was considered representative of the entire population; according to the Taiwan National Health Research Institute, the group did not differ statistically from the larger cohort in age, sex, or health care costs [13, 14]. This sample was used as our study cohort.

### Study population

**Fig. 1:**
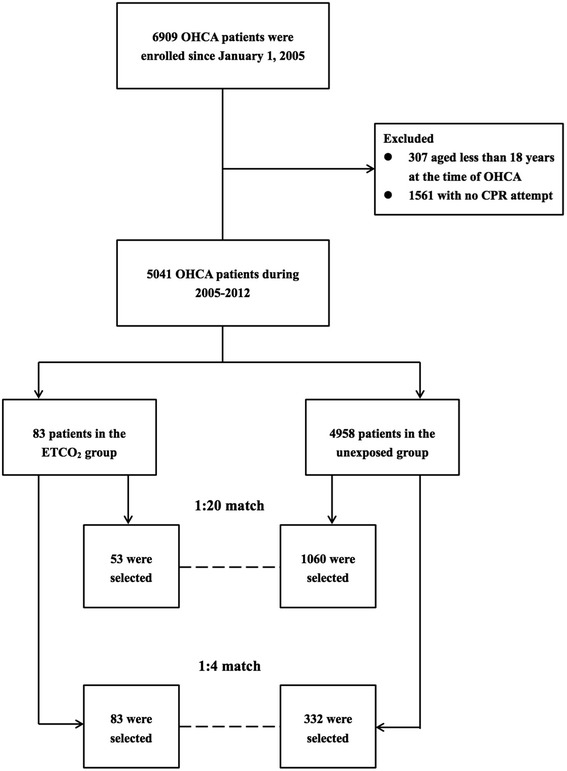
Flow diagram of the population-based study

The population sample was followed from 1 January 2003 to 31 December 2012 (a total of 10 years). For our study cohort, we first identified individuals who were still alive in 2005 and were older than 18 years at the time of OHCA. OHCA was defined by the ICD-9-CM codes ventricular fibrillation (427.4), cardiac arrest (427.5), and sudden death (798.0–798.9) in outpatient clinic records. To avoid including patients who had Do Not Attempt Resuscitation orders, no chance of survival, and coding errors, we excluded patients on whom no chest compression was attempted. ETCO_2_ monitoring was defined by the charge record for continuous capnography during the visits. For each charge record of ETCO_2_, the institutes could claim for about US$ 14 from the Bureau of National Health Insurance. After exclusions, we identified 83 patients who received ETCO_2_ monitoring and 4958 who did not receive such monitoring. In order to identify sustained ROSC, each patient was tracked if he or she was hospitalized after OHCA (Fig. 1).

### Prespecified covariates

In order to investigate a significant influence on survival associated with ETCO_2_-monitoring, we included several covariates in the analysis; age, sex, calendar year, urbanization level (i.e., urban, suburban, and rural), health care institutes, and socioeconomic status (SES). Income-related insurance payment amounts were used as a proxy measure of individual SES at follow-up. People were divided into 3 groups: (1) low SES: payment lower than US$571 per month (New Taiwan Dollars [NT$] 20,000); (2) moderate SES: payment between US$571 and US$1141 per month (NT$ 20,000–40,000); and (3) high SES: payment of US$1142 or more per month (NT$40,001 or more) [12]. The health care institutes visited by patients were classified into 4 levels (medical centers, regional hospitals, local hospitals, and clinics) based on hospital accreditation. Two additional covariates that may be related to sustained ROSC following resuscitation, the CPR duration and attempted defibrillation, were identified based on charge records. Finally, the prevalence of selected comorbid conditions (i.e., diabetes, hypertension, coronary artery disease, hyperlipidemia, malignancies, heart failure, atrial fibrillation, intracranial hemorrhage, ischemic stroke, chronic renal insufficiency, and liver cirrhosis) and the Charlson Comorbidity Index (CCI) score were determined using discharge diagnoses either during outpatient clinic visits or hospitalizations before 1 January 2005. The CCI is a scoring system that assigns weights to important concomitant diseases; it has been validated for use in studies that employ ICD-9-CM data [14, 15].

### Propensity score methods

In this study, the propensity score was the conditional probability for using ETCO_2_ monitoring in the presence of possible confounders. The prespecified covariates were added into a multivariable logistic regression model to predict the probability of ETCO_2_ use. The predicted probability from the model was used as the propensity score for each patient. We then matched each patient in the ETCO_2_ group to 20 patients in the untreated group with the closest propensity score using a standard greedy-matching algorithm [16] and compared the probability of survival benefits between these groups.

### Statistical analysis

The SAS statistical package, version 9.4 (SAS Institute, Inc., Cary, NC, USA) was used for data analysis. All covariates were taken as categorical variables except age, calendar year, CPR duration, and propensity score, which were treated as continuous variables. Categorical variables were compared using Pearson’s chi-square test, and continuous variables were assessed using the *t* test to determine baseline heterogeneity in the 2 groups. Simple conditional logistic regression models were then used to calculate the ORs of sustained ROSC and survival to hospital discharge for patients with ETCO_2_ use in the matched group.

In order to further assess the robustness of our results, we sampled another cohort by matching each patient in the ETCO_2_ group to 4 patients in the untreated group using the same method (Fig. 1). We compared the crude ORs and risk differences of survival benefits among 2 matched cohorts and the original group to evaluate if the results are similar. We also evaluated the extent of the effect of a potentially unmeasured confounder in accounting for the results [17]. A two-tailed *P* value of 0.05 was considered significant.

## Results

The distribution of demographic characteristics and selected morbidities in both groups is shown in Table 1. There were 6909 episodes of OHCA during the 8-year period. The population incidence of OHCA in Taiwan was 86.3 per 100,000 person-years. After exclusion, there were 83 patients in the ETCO_2_ group and 4958 in the untreated group. The overall baseline characteristics are similar between the 2 groups, except the patients monitored with ETCO_2_ were more likely to have liver cirrhosis. In addition, patients who visited medical centers also had a higher probability of receiving ETCO_2_ monitoring. The proportion of ETCO_2_ use gradually increased after 2010 but still was low. In 2012, only 5.8 % of OHCA patients received ETCO_2_ monitoring (Table 2). Detailed information about the frequency of ETCO_2_ use are summerized in the Additional file 1. After resuscitation, 658 patients (13 %) had records of sustained ROSC, including 25 patients in the ETCO_2_ group and 633 in the untreated group. Among them, 1 patient (4 %) in the ETCO_2_ group and 96 (15.2 %) in the untreated group had a record of survival to hospital discharge, respectively. The crude odds ratio (OR) of sustained ROSC for the ETCO_2_ group was 2.95 (95 % CI 1.83–4.74).

**Table 1:** Baseline characteristics of the ETCO_2_ group and the untreated group

Variables	ETCO_2_ group		Untreated group		*P* value
	(*n* = 83)		(*n* = 4958)		
Male, no. (%)	44	53.0	3138	63.3	0.054
Mean age in years (SD)	71.3	15.2	67.9	17.6	0.088
Attempted defibrillation, no. (%)	14	16.9	878	17.7	0.842
Mean CPR time (10 min) (SD)	2.4	1.4	2.4	1.6	0.927
Socioeconomic status, no. (%)					0.236
Low	56	67.5	3586	72.3	
Moderate	26	31.3	1212	24.5	
High	1	1.2	160	3.2	
Urbanization level, no. (%)					0.118
Urban	26	31.3	1174	23.7	
Suburban	29	35.0	2259	45.6	
Rural	28	33.7	1525	30.7	
Diabetes, no. (%)	25	30.1	1255	25.3	0.318
Hypertension, no. (%)	43	51.8	2434	49.1	0.624
Coronary artery disease, no. (%)	16	19.3	1075	21.7	0.598
Hyperlipidemia, no. (%)	21	25.3	893	18.0	0.087
Malignancies, no. (%)	6	7.2	231	4.7	0.273
Heart failure, no. (%)	1	1.2	255	5.1	0.105
Atrial fibrillation, no. (%)	3	3.6	107	2.2	0.368
Charlson comorbidity index score, no. (%)					0.821
0	25	30.1	1631	32.9	
1	24	28.9	1307	26.4	
≥ 2	34	41.0	2020	40.7	
Intracerebral hemorrhage, no. (%)	1	1.2	108	2.2	0.545
Ischemic stoke, no. (%)	16	19.3	887	17.9	0.744
Chronic renal insufficiency, no. (%)	2	2.4	233	4.7	0.326
Liver cirrhosis, no. (%)	16	19.3	550	11.1	0.019
Health care institutes, no. (%)					<0.001
Medical centers	36	43.4	1108	22.3	
Regional hospitals	41	49.4	2497	50.4	
Local hospitals	6	7.2	1333	26.9	
Clinics	0	0	20	0.4	
Mean propensity score (SD)	0.046	0.041	0.016	0.021	<0.001

**Table 2:** Frequency of ETCO_2_ use

	Frequency of ETCO_2_ use			
Year	ETCO_2_ use		Untreated group	
	NO.	%	NO.	%
2005	7	1.32	522	98.68
2006	2	0.35	571	99.65
2007	5	0.86	574	99.14
2008	7	1.04	666	98.96
2009	0	0	643	100
2010	7	1.07	646	98.93
2011	14	2.03	675	97.97
2012	41	5.84	661	94.16

Next, 53 patients in the ETCO_2_ group and 1060 in the untreated group were selected after propensity score matching algorithm. In the subcohort, 165 patients survived, including 15 in the ETCO_2_ group and 150 in the untreated group. Among them, 1 patient (6.7 %) in the ETCO_2_ group and 22 (14.7 %) in the untreated group had a record of survival to hospital discharge, respectively. The basic characteristics of these 2 subgroups are summarized in Table 3. After matching, all baseline characteristics were similar between the 2 groups. A simple conditional logistic regression model was used to estimate the OR for sustained ROSC and remained significantly higher in patients with ETCO_2_ use (2.38, 95 % CI 1.28–4.42, *P* = 0.006). A simple conditional logistic regression model was again used to estimate the OR of survival to hospital discharge but failed to find treatment benefit regarding ETCO_2_ use (OR 0.91, 95 % CI 0.12–6.90 *P* = 0.924).

**Table 3:** Baseline characteristics in the propensity-matched cohort

Variables	ETCO_2_ group		Untreated group		*P* value
	(*n* = 53)		(*n* = 1060)		
Male, no. (%)	27	50.9	644	60.8	0.154
Mean age in years (SD)	68.9	15.9	69.6	16.7	0.776
Attempted defibrillation, no. (%)	11	20.8	183	17.3	0.513
Mean CPR time (10 min) (SD)	2.7	1.4	2.5	1.6	0.394
Socioeconomic status, no. (%)					0.864
Low	38	71.7	727	68.6	
Moderate	14	26.4	316	29.8	
High	1	1.9	17	1.6	
Urbanization level, no. (%)					0.929
Urban	13	24.5	276	26.0	
Suburban	23	43.4	469	44.3	
Rural	17	32.1	315	29.7	
Diabetes, no. (%)	11	20.8	272	25.7	0.424
Hypertension, no. (%)	26	49.1	521	49.2	0.989
Coronary artery disease, no. (%)	10	18.9	200	18.9	1.000
Hyperlipidemia, no. (%)	10	18.9	211	19.9	0.853
Malignancies, no. (%)	3	5.7	44	4.2	0.594
Heart failure, no. (%)	1	1.9	19	1.8	0.959
Atrial fibrillation, no. (%)	1	1.9	16	1.5	0.827
Charlson Comorbidity Index score, no. (%)					0.935
0	19	35.9	355	33.5	
1	15	28.3	305	28.8	
≥ 2	19	35.9	400	37.7	
Intracerebral hemorrhage, no. (%)	1	1.9	26	2.5	0.794
Ischemic stoke, no. (%)	8	15.1	198	18.7	0.512
Chronic renal insufficiency, no. (%)	2	3.8	36	3.4	0.883
Liver cirrhosis, no. (%)	4	7.6	128	12.1	0.320
Health care institutes, no. (%)					0.959
Medical centers	15	28.3	288	27.2	
Regional hospitals	32	60.4	639	60.3	
Local hospitals	6	11.3	133	12.6	
Clinics	0.0209	0.0125	0.0207	0.0125	0.917

**Fig. 2:**
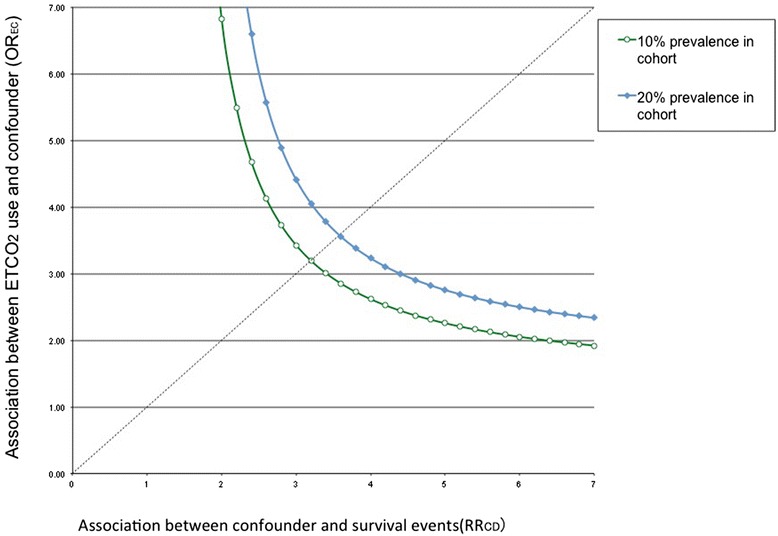
Sensitivity analyses for an unmeasured confounding factor

Crude ORs and risk differences among the original group and two matched cohorts were found to be similar. The results are summarized in Table 4. Sensitivity analyses showed that an unmeasured confounder present in 10 % of the study population would be required to elevate the possibility of sustained ROSC by a factor of 3.2. Among patients with ETCO_2_ use, the confounder also would have to be approximately 3.2 times more prevalent than that among the untreated group in order to explain a lower 95 % confidence limit HR of 1.28 (Fig. 2).

**Table 4:** Comparison of ORs and risk differences among the original group and matched cohorts

		ETCO2 group (*n*)	Untreated group (*n*)	ORs (95 % CI)	Risk differences (95 % CI)
Original group	ROSC(+)	25	633	2.95 (1.83–4.74)	0.17 (0.07–0.27)
	ROSC(−)	58	4325		
1:4 matched cohort	ROSC(+)	25	56	2.12 (1.23–3.68)	0.13 (0.02–0.23)
	ROSC(−)	58	276		
1:20 matched cohort	ROSC(+)	15	150	2.39 (1.29–4.46)	0.14 (0.02–0.26)
	ROSC(−)	38	910

## Discussion

The ACLS guideline for using ETCO_2_ monitoring during CPR provides the basis on which providers can have real-time feedback about the quality of chest compressions, thus offering patients a better chance of survival [18, 19]. To our knowledge, ours is the first study regarding possible survival benefits with ETCO_2_ monitoring in real clinical situations. In this study, we evaluated the sustained ROSC of patients in Taiwan who received ETCO_2_ monitoring following OHCA. The database corresponded well to the characteristics of the whole population; therefore, loss of follow-up or selection bias were not concerns. Although the overall survival rates of OHCA patients in Asia were found to be low [20], patients with ETCO_2_ monitoring during resuscitation still had a higher possibility of sustained ROSC (OR 2.38, 95 % CI 1.28–4.42). Although there were relatively few patients in the treatment group, based on our sample size of matched cohort and ORs the statistical power still achieved 1.0.

ETCO_2_ monitoring by itself cannot directly improve the quality of CPR administered. However, based on the real-time feedback about the patient’s ETCO_2_ level, the code team can adjust their management to achieve better chest compression quality, which may in turn result in a better outcome. Moreover, ETCO_2_ monitoring may also serve as a proxy for better team performance because teams or institutes that use ETCO_2_ monitoring may have better insights about the importance of CPR performance and thus may have better outcomes. Increased CPR quality may increase the probability of the return of spontaneous circulation. However, in the absence of data linking ETCO_2_ measurements with the quality markers of chest compressions in our study, no causality inference can be drawn currently.

This study is also the first one based on national data about the use of capnography during OHCA. Despite of the strong recommendation for its use, ETCO_2_ monitoring in OHCA patients is still low in Taiwan. Even in 2012, the overall percentage of ETCO_2_ monitoring was only 5.84 %. According to the recommendations of the ACLS guidelines and the possible survival benefits found in this study, further emphasis should be placed on the routine use of ETCO_2_ monitoring during OHCA.

### Limitations

First, our findings were generated from administrative data. The definitions of OHCA were based on ICD-9-CM codes, which are useful for insurance reimbursement but may not be exact substitutes for precise operative definitions. There is also lack of data whether ETCO_2_ monitoring was performed during or after chest compressions. We validated the selection processes by analyzing 150 medical records of patients with OHCA randomly selected from the electronic database from 2010 to 2012 at Dalin Tzu Chi Hospital, Buddhist Tzu Chi Medical Foundation. All 118 patients who were charged with chest compression had documented CPR records. Among 69 patients with ETCO_2_ charge records, the confirmation of use was found by documented CO_2_ levels during resuscitations. All 42 patients with records of hospitalization were alive before admissions. These selection processes yielded positive predictive values of 100 %.

Second, the percentage of ETCO_2_ monitoring in our study cohort was only 1.6 %, which may be an underestimate of the true number of patients who actually were monitored. In clinical practice in Taiwan, procedures are charged either by healthcare or administrative staff. In a busy situation such as resuscitation, staff may forget to charge for the ETCO_2_ monitoring in some patients. Although the extent of crossover to the treatment category cannot be assessed in this study, according to the intention-to-treat analytical principle, the result in this study would show only a bias toward a null result, and the estimation of OR would be more conservative than the actual number reported.

Third, the overall percentage of sustained ROSC (13 %) is low compared with results reported in other publications [20, 21]. Results similar to ours were found in a study published by Huang et al. [22], and fewer shockable rhythms in that study may account for the similarity. Because of the limited number of cases, we failed to find benefits of ETCO_2_ monitoring on patients’ survival to hospital discharge. Further study should be conducted to evaluate if chest compression monitoring by ETCO_2_ can improve survival to hospital discharge and, perhaps, if it can be associated with better cerebral performance.

Fourth, for an observational study, confounding by indication may be a concern even after one applies propensity score matching to establish the comparability between groups that use ETCO_2_ monitoring or do not. For example, in patients with a lower likelihood of survival, physicians may be less likely to use ETCO_2_ monitoring during resuscitation. However, because the outcome of survival is not perfectly predictable beforehand, such an argument is not plausible. Moreover, because the baseline characteristics were similar between the ETCO_2_ group and the untreated group even before matching, we believe that resuscitation teams would use ETCO_2_ monitoring guidelines instead of clinical judgments.

Fifth, with the use of propensity score analyses to adjust for possible confounding, we also lost the ability to evaluate other possible covariates of survival. Finally, although we extensively adjusted for many possible covariates, unmeasured confounding and the possibility of overmatching may still exist. In our database, we were unable to obtain prehospital information such as bystander CPR, prehospital intubation, and duration of collapse.

## Conclusion

Patients monitored with ETCO_2_ may have a higher possibility of sustained ROSC. However, the overall use of ETCO_2_ is still low despite strong recommendations in guidelines.

## Additional file

Additional file 1:
**Frequency of ETCO2 use. **(DOCX 28 kb)

## Abbreviations

ACLS: Advanced cardiac life support
CCI: Charlson comorbidity index
CPR: Cardiopulmonary resuscitation
ETCO_2_: End-tidal carbon dioxide monitoring
ICD-9-CM: International Classification of Diseases (Ninth Revision) Clinical Modification
NHI: National Health Insurance
OHCA: Out-of-hospital cardiac arrest
OR: Odds ratio
SES: Socioeconomic status
