# Supplementary material for: End-tidal carbon dioxide monitoring may be associated with a higher possibility of return of spontaneous circulation during out-of-hospital cardiac arrest: a population-based study
Source: Scand J Trauma Resusc Emerg Med. 2015 Nov 24;23:104. doi: 10.1186/s13049-015-0187-y (PMC4657353; doi:10.1186/s13049-015-0187-y)
Supplement: Additional file 1: — Frequency of ETCO2 use. (DOCX 28 kb) [file 13049_2015_187_MOESM1_ESM.docx]

|  | Frequency of ETCO2 use | | | | | | | | | | | | | | | |
| --- | --- | --- | --- | --- | --- | --- | --- | --- | --- | --- | --- | --- | --- | --- | --- | --- |
| **Year**  **Variables** | 2005 | | 2006 | | 2007 | | 2008 | | 2009 | | 2010 | | 2011 | | 2012 | |
|  | NO. | % | NO. | % | NO. | % | NO. | % | NO. | % | NO. | % | NO. | % | NO. | % |
| Male (n=3182) | 3 | 0.89 | 1 | 0.26 | 2 | 0.54 | 2 | 0.48 | 0 | 0 | 5 | 1.22 | 6 | 1.49 | 25 | 5.54 |
| Attempted defibrillation (n=892) | 1 | 0.82 | 0 | 0 | 0 | 0 | 1 | 0.86 | 0 | 0 | 2 | 1.71 | 2 | 1.64 | 8 | 7.84 |
| CPR time less than 10 minutes (n=2360) | 2 | 0.65 | 0 | 0 | 3 | 1.06 | 3 | 1.06 | 0 | 0 | 3 | 1.03 | 4 | 1.29 | 14 | 4.88 |
| CPR time more than 10 minutes (n=2681) | 5 | 2.28 | 2 | 0.79 | 2 | 0.68 | 4 | 1.02 | 0 | 0 | 4 | 1.10 | 10 | 2.64 | 27 | 6.51 |
| Socioeconomic status |  |  |  |  |  |  |  |  |  |  |  |  |  |  |  |  |
| High (n= 161) | 0 | 0 | 0 | 0 | 0 | 0 | 0 | 0 | 0 | 0 | 1 | 5.26 | 0 | 0 | 0 | 0 |
| Others (n=4880) | 7 | 1.36 | 2 | 0.36 | 5 | 0.89 | 7 | 1.07 | 0 | 0 | 6 | 0.95 | 14 | 2.11 | 86 | 6.12 |
| Urbanization level |  |  |  |  |  |  |  |  |  |  |  |  |  |  |  |  |
| Urban (n=1200) | 0 | 0 | 0 | 0 | 3 | 2.17 | 2 | 1.23 | 0 | 0 | 1 | 1.89 | 4 | 2.33 | 14 | 7.87 |
| Others (n=3841) | 7 | 1.72 | 2 | 0.44 | 2 | 0.45 | 5 | 0.98 | 0 | 0 | 4 | 0.81 | 10 | 1.93 | 27 | 5.15 |
| Liver cirrhosis (n=566) | 0 | 0 | 1 | 1.43 | 0 | 0 | 2 | 0 | 0 | 0 | 0 | 0 | 3 | 3.8 | 10 | 11.36 |
| Diabetes (n=1280) | 3 | 2.31 | 1 | 0.70 | 1 | 0.64 | 0 | 0 | 0 | 0 | 2 | 1.16 | 2 | 1.18 | 16 | 9.36 |
| Hypertension (n=2477) | 3 | 1.08 | 0 | 0 | 4 | 1.35 | 3 | 0.90 | 0 | 0 | 6 | 1.81 | 6 | 1.88 | 21 | 6.44 |
| Hyperlipidemia (n=914) | 3 | 3.37 | 0 | 0 | 2 | 1.79 | 1 | 0.87 | 0 | 0 | 3 | 2.31 | 4 | 2.86 | 8 | 5.97 |
| Coronary artery disease (n=1091) | 3 | 2.22 | 0 | 0 | 1 | 0.68 | 1 | 0.71 | 0 | 0 | 2 | 1.47 | 3 | 2.38 | 6 | 4.14 |
| Malignancies (n=237) | 1 | 2.86 | 1 | 3.57 | 1 | 4.00 | 0 | 0 | 0 | 0 | 0 | 0 | 1 | 3.23 | 2 | 6.90 |
| Intracerebral hemorrhage (n=109) | 0 | 0 | 0 | 0 | 0 | 0 | 0 | 0 | 0 | 0 | 1 | 5.56 | 0 | 0 | 0 | 0 |
| Charlson Comorbidity Index score |  |  |  |  |  |  |  |  |  |  |  |  |  |  |  |  |
| ≥ 2(n=2054) | 5 | 1.91 | 1 | 0.41 | 3 | 1.15 | 2 | 0.71 | 0 | 0 | 4 | 1.62 | 5 | 1.89 | 14 | 5.98 |
| 0 or 1(n=2987) | 2 | 0.75 | 1 | 0.30 | 2 | 0.63 | 5 | 1.28 | 0 | 0 | 3 | 0.74 | 9 | 2.12 | 27 | 5.77 |
| Cerebral vascular accident (n=903) | 1 | 0.77 | 1 | 1.01 | 1 | 0.84 | 2 | 1.49 | 0 | 0 | 3 | 2.63 | 2 | 1.80 | 6 | 6.38 |
| heart failure(n=256) | 0 | 0 | 0 | 0 | 0 | 0 | 0 | 0 | 0 | 0 | 1 | 3.13 | 0 | 0 | 0 | 0 |
| Atrial fibrillation (n=110) | 1 | 7.14 | 0 | 0 | 0 | 0 | 1 | 6.25 | 0 | 0 | 1 | 7.69 | 0 | 0 | 0 | 0 |
| Chronic Renal Insufficiency (n=235) | 1 | 3.23 | 0 | 0 | 0 | 0 | 0 | 0 | 0 | 0 | 0 | 0 | 0 | 0 | 1 | 5.56 |
| Health care institutes |  |  |  |  |  |  |  |  |  |  |  |  |  |  |  |  |
| medical center(n=1150) | 5 | 3.29 | 0 | 0 | 2 | 1.48 | 2 | 1.37 | 0 | 0 | 6 | 4.58 | 4 | 2.58 | 17 | 11.56 |
| Others(n=3891) | 2 | 0.53 | 2 | 0.47 | 3 | 0.68 | 5 | 0.95 | 0 | 0 | 1 | 0.19 | 10 | 1.87 | 24 | 4.32 |
